# Without a silhouette

**DOI:** 10.1002/ccr3.3469

**Published:** 2020-11-21

**Authors:** Yosuke Sazumi, Kazuki Tokumasu, Hideharu Hagiya, Fumio Otsuka

**Affiliations:** ^1^ Department of General Medicine Okayama University Graduate School of Medicine Dentistry and Pharmaceutical Sciences Okayama Japan

**Keywords:** pleural effusion, primary effusion lymphoma

## Abstract

Primary effusion lymphoma (PEL) is a kind of malignant lymphoma that develops without a tumor mass. A fluid smear is of no use for the diagnosis of miscellaneous condition. Repeated cell‐block smear with immunostaining is useful for the diagnosis of PEL.

## Graphical abstract

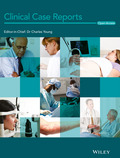

## 1 MANUSCRIPT

**FIGURE 1:**
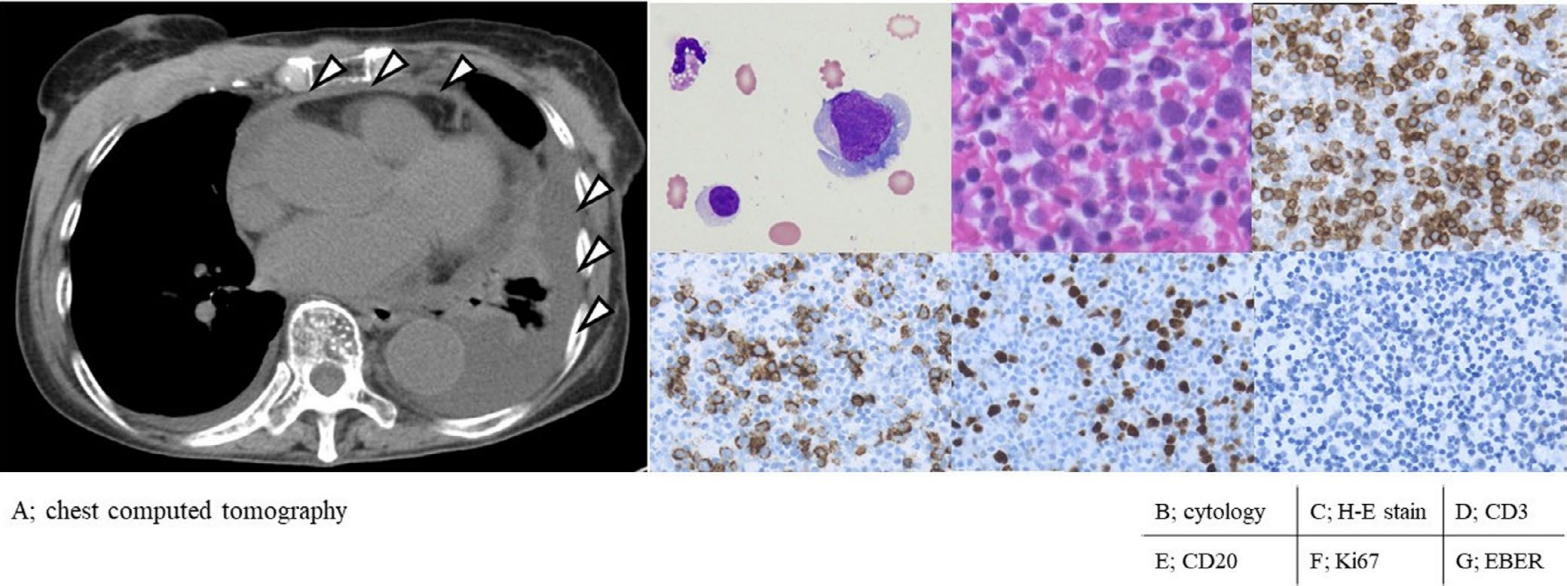
Chest computed tomography and pathological findings of pleural effusion. Cardiac and pleural effusions (A). Atypical lymphoid cells were identified in the pleural effusion by cytology (B) and cell‐block smear (C) (hematoxylin and eosin stain). Immunostaining revealed CD3 (D), CD20 (E), and Ki‐67 (F) positivity and EBV‐encoded small RNA (EBER, G) negativity. CD, cluster of differentiation; EBV, Epstein‐Barr virus

An 84‐year‐old woman was admitted to our hospital because of worsening dyspnea and persistent fever. Her serum C‐reactive protein and soluble interleukin‐2 receptor levels were elevated as 18.57 U/L and 2113 U/mL, respectively. Chest computed tomography revealed pleural and cardiac effusions (Figure 1A). Pleurocentesis showed a pale‐bloody, yellowish exudative effusion, with findings of increased cells (monocyte‐dominant 880 cells/μL), elevated total protein (4.2 g/dL), and a presence of atypical lymphoid cells (Figure 1B). A cell‐block smear of the pleural effusion showed aggregation of CD‐3‐positive, CD‐20‐positive, Ki67‐positive, and EBV‐encoded small RNA (EBER)‐negative abnormal cells (Figure 1C‐G). Reactivation of human herpesvirus (HHV)‐8 was not confirmed due to the unavailability of the test in our setting. What is your diagnosis?

## 2 ANSWER

The patient was diagnosed with primary effusion lymphoma (PEL)‐like lymphoma. PEL is a rare non‐Hodgkin's lymphoma that is classified as a mature B‐cell neoplasm, which is associated with HHV‐8 infection and lacks CD20 expression because of plasmablastic differentiation.^1^, ^2^, ^3^ In case HHV‐8 reactivation is not confirmed or related as this case, it is considered PEL‐like lymphoma. Differential diagnosis of the disease includes plasmablastic lymphoma or pyothorax‐associated lymphoma. A fluid smear is often of no use for the miscellaneous condition, whereas a cell‐block smear and immunostaining procedures potentially lead to a diagnosis.

## CONFLICT OF INTEREST

The authors state that there are no conflicts of interests to declare.

## AUTHOR CONTRIBUTIONS

YS, KT, HH, and FO have contributed to the management of the patient and drafting of the manuscript.
